# First Comprehensive Analysis of Full-Length and Δ2 Foxp3 Isoforms Distribution in PBMCs from Healthy Volunteers

**DOI:** 10.3390/biom16070948

**Published:** 2026-06-25

**Authors:** Manuel Fernández-Delgado, Luis Sendra, María José Herrero, Gladys G. Olivera-Pasquini, Enrique G. Zucchet, Raimundo García-Boyero, Salvador F. Aliño

**Affiliations:** 1 Service of Hematology and Hemotherapy, Hospital General Universitario de Castellón, 12004 Castelló de la Plana, Spain; fernandez-delgado_man@gva.es (M.F.-D.); garcia_rai@gva.es (R.G.-B.); 2 Gene Therapy and Pharmacogenomics, Department of Pharmacology, Universitat de València, 46010 Valencia, Spain; maria.jose.herrero@uv.es; 3 Unit of Pharmacogenetics and Gene Therapy, La Fe Health Research Institute, 46026 Valencia, Spain

**Keywords:** FOXP3, FOXP3 isoforms, regulatory T cells, flow cytometry, PD-1, CTLA-4, immune checkpoints, leukocyte subsets

## Abstract

FOXP3 is the master transcriptional regulator of regulatory T cells (Tregs) and is expressed in humans as two main alternatively spliced isoforms: full-length FOXP3 (FOXP3-FL) and the exon 2-deficient variant (FOXP3-Δ2). While the role of these isoforms has been mainly studied in CD4^+^ T cells, their distribution across peripheral blood leukocyte populations and their relationship with immune checkpoint expression remain incompletely defined. In this study, we used a multiparametric flow cytometry panel allowing isoform-specific detection of FOXP3-FL and FOXP3-Δ2, together with PD-1 and CTLA-4, to analyze peripheral blood samples from six healthy donors under basal conditions. Major leukocyte populations, including CD4^+^CD25^+^ and CD4^+^CD25^−^ T cells, CD8^+^ T cells, monocytes, and neutrophils, were evaluated. FOXP3-FL predominated in CD4^+^CD25^+^ T cells, whereas FOXP3-Δ2 was more frequently detected in CD8^+^ T cells, monocytes, and neutrophils. However, the absolute frequencies of these FOXP3-Δ2-positive populations were low, consistent with the overall low levels of FOXP3 expression observed in these cell types. In CD4^+^ T-cell subsets, PD-1 expression was generally higher than CTLA-4, regardless of FOXP3 isoform, and FOXP3-Δ2^+^ cells showed relatively higher PD-1 expression compared to FOXP3-FL^+^ cells. In contrast, checkpoint expression in non-CD4^+^ populations was limited. The observed FOXP3-FL^+^/FOXP3-Δ2^+^ ratios across immune cell populations were consistent with a predominant role of FOXP3-FL in maintaining immune tolerance under basal conditions; whether these patterns are preserved or altered in pathological settings warrants further investigation. These results provide a descriptive overview of FOXP3 isoform distribution and checkpoint expression across peripheral blood immune cell subsets in healthy individuals, which may serve as a reference for future studies in immune-mediated diseases.

## 1. Introduction

Regulatory T lymphocytes (Tregs) are an essential lymphocyte subset for immune homeostasis [1], critically suppressing immune responses and preventing autoimmunity. Beyond immune regulation, Tregs contribute to tissue remodeling and play decisive roles in shaping the tumor microenvironment [2]. Their suppressive activity is mediated through multiple mechanisms, including the trogocytosis of antigen-presenting cells or target cells via cytotoxic T lymphocyte antigen 4 (CTLA-4) [3], the secretion of immunoregulatory cytokines, and the sequestration of IL-2 [4,5,6]. Within this regulatory axis, the transmembrane protein programmed cell death 1 (PD-1) acts as a key inhibitory receptor expressed on T, B, and NK lymphocytes as well as monocytes, functioning as an immune checkpoint that fine-tunes T-cell activity, function, and tolerance [7].

Treg cells are identified by the expression of the FOXP3 protein. FOXP3 is considered the master transcription factor of regulatory T (Treg) cells, orchestrating a transcriptional program required for their development, stability, and suppressive function. The FOXP3 gene, which is found on the X chromosome (positions 49,250,436–49,264,826 in the GRCh38 assembly), codes for the forkhead box P3 (FOXP3) transcription factor. FOXP3 is a lineage-specifying “master regulator” that orchestrates the Treg suppressive program [8]. Its expression is regulated by a complex network of signaling pathways involving T-cell receptor activation, IL-2/STAT5 signaling, TGF-β-mediated pathways, epigenetic modifications, and enhancer elements within the FOXP3 locus [9]. The gene comprises 11 coding exons and, through alternative splicing, generates multiple isoforms in humans. The two most prevalent isoforms are the full-length isoform (FOXP3-FL) and the exon-2-deficient isoform (FOXP3-ΔE2). The exact molecular mechanism of the alternative splicing of Foxp3 pre-mRNA is unknown. However, T-cell receptor (TCR) activation can significantly promote Foxp3Δ2 formation [10]. FOXP3 splicing isoforms show distinct abilities in nuclear translocation, DNA binding, and cofactor interaction, resulting in different responses in differentiation, cytokine secretion, suppressive function, and lineage stability of Tregs [11]. The region encoded by exon 2 harbors distinct protein-interaction domains and a nuclear export signal that collectively contribute to the regulation of FOXP3 transcriptional activity and Treg lineage stability [12,13,14]. The loss of FOXP3 exon 2 can also enhance DNA-binding ability [11]. Consistent with this, murine studies have demonstrated that Tregs exclusively expressing the FOXP3-ΔE2 isoform exhibit intrinsic instability, with progressive loss of FOXP3 expression in vivo and cell-intrinsic reductions in key Treg identity markers, including CD25 and CTLA-4 [15]. It has also been shown that the expression of FOXP3-Δ2 is associated with the promotion of CD8 T-cell-mediated antitumor immunity, thus providing resistance to the development of multiple types of tumors [16]. Increased or imbalanced expression of ΔE2 in relation to FL is often linked to Treg dysfunction and autoimmunity.

Regulatory T cells exert suppressive functions through multiple mechanisms, including the expression of immune checkpoint molecules such as CTLA-4, PD-1, TIGIT, and LAG-3, many of which are directly or indirectly regulated by FOXP3 [17,18]. Besides the suppressive role of FOXP3 in canonical CD4+CD25+ Treg cells, experimental studies have further demonstrated that ectopic expression of FOXP3 in conventional CD4+CD25− T cells can confer suppressive properties and induce the expression of several Treg-associated markers (GITR, CTLA-4, and TGFβ), suppress the proliferation of naïve CD4+T cells, and inhibit activation-induced IL-2 gene expression and the production of IFNϒ in response to TCR ligation [19], thus supporting its central role in establishing the regulatory T-cell phenotype. CTLA-4 and PD-1 play complementary and linked but temporally and spatially distinct roles in immune suppression. CTLA-4 is constitutively expressed in Tregs and plays an essential role in their suppressive function by limiting CD28-mediated co-stimulation of naïve T cells and increasing free PD-L1 availability through a cell-contact-dependent mechanism [3,20,21]. CTLA-4 expression has been previously explored in relation to FOXP3 isoforms in murine lymphoid tissues [10] and in FOXP3-transduced human CD4^+^CD25^−^ T cells [22]. In contrast, PD-1 is induced upon T-cell receptor (TCR) stimulation and mainly regulates T-cell activity during the effector phase in peripheral tissues by inhibiting CD28 and TCR signaling pathways [21,23]. PD-1-mediated inhibitory signaling is more sustained and functionally effective [24] and, under conditions of persistent antigenic stimulation, can contribute to the development of T-cell exhaustion [23]. Despite these well-established roles, the relationship between CTLA-4 and PD-1 expression and FOXP3 isoform identity remains unexplored in human peripheral blood under basal physiological conditions.

Previous flow cytometry studies have quantified FOXP3-FL and FOXP3-Δ2 expression in Tregs from various diseases compared with healthy donors [9,10]. However, no quantitative data exist for other peripheral blood cell subsets (CD8^+^ T cells, neutrophils, and monocytes). Likewise, although CTLA-4 and PD-1 expression have been extensively characterized [25,26,27], little is known about their distribution across immune cell subsets defined by FOXP3 isoform expression. Given the increasing clinical relevance of immune checkpoint modulation in cancer, autoimmunity, and chronic inflammatory diseases, understanding the distribution of FOXP3 isoforms across immune cell populations may provide valuable insights into mechanisms of immune regulation and potential biomarkers of immune dysfunction.

Here, we developed a multiparametric flow cytometry panel to quantify FOXP3-FL and FOXP3-Δ2 in peripheral blood from healthy donors under basal conditions, not only in Tregs but also in CD4^+^/CD25^−^ T cells, CD8^+^ T cells, monocytes, and neutrophils, using an isoform-specific antibody clone for FOXP3-Δ2. We further examined PD-1 and CTLA-4 expression within these populations.

The primary objective was to define the baseline distribution of the two predominant FOXP3 isoforms (FOXP3-FL and FOXP3-Δ2) across peripheral blood immune subsets in healthy donors and to assess the expression of the inhibitory receptors PD-1 and CTLA-4 within these subsets.

## 2. Materials and Methods

### 2.1. Human Blood Samples

Peripheral blood samples were obtained from six healthy donors at the Castelló Blood Transfusion Center after provision of study information and signing of informed consent. No data on donor age or sex were collected. Samples were collected in EDTA tubes, and experiments were initiated within 2 h of collection.

### 2.2. Reagents

All the monoclonal antibodies were purchased from BD Biosciences, San Jose, CA, USA. The monoclonal antibodies used were: CD4 (clone SK3, PerCP-Cy™5.5), CD8 (clone SK1, APC-H7), CD152/CTLA-4 (clone BNI3, BV421), CD25 (clone 2AR, PE-Cy™7), CD279/PD-1 (clone MIH4, APC), FOXP3 (clone 236A/E7), and Fixable Viability Stain 510 (BD Biosciences, San Jose, CA, USA). Detection of the FOXP3-Δ2 isoform was performed using an in-house biotin-conjugated antibody (clone 16J4G6, Invitrogen, Carlsbad, CA, USA). Biotinylating was carried out using the Pierce™ Antibody Biotinylating Kit for IP (Thermo Fisher Scientific, Rockford, IL, USA) according to the manufacturer’s instructions. Antibodies targeting FOXP3 (15 µL), FOXP3-Δ2 (15 µL), CD4 (10 µL), CD25 (5 µL), CD152 (5 µL), CD279 (5 µL), and the viability marker (0.5 µL) were titrated following the manufacturer’s recommendations to determine optimal concentrations; CD8 was used at manufacturer-recommended dilutions.

### 2.3. Flow Cytometry

For each experiment, 500 μL of whole peripheral blood was used. Red blood cells were lysed by adding 4 mL of BD Pharm Lyse™ buffer (BD Biosciences, San Jose, CA, USA). The suspension was centrifuged, and the pellet was washed with PBS containing 1% heat-inactivated fetal bovine serum and 0.1% sodium azide (PBS–FBS), followed by centrifugation at 200× *g* for 10 min. A second lysis step using the same lysis buffer was performed if necessary.

Cells were first incubated with the viability dye following the manufacturer’s recommended protocol and then stained with surface antibodies (CD4, CD8, PD-1, CTLA-4, and CD25). Intracellular staining was performed using the BD Pharmingen™ Human FOXP3 Buffer Set (BD Biosciences) for fixation, permeabilization, and detection of both FOXP3 isoforms, according to the manufacturer’s instructions. Streptavidin was subsequently added to detect the biotin-conjugated anti-FOXP3-Δ2 antibody.

**Figure 1:**
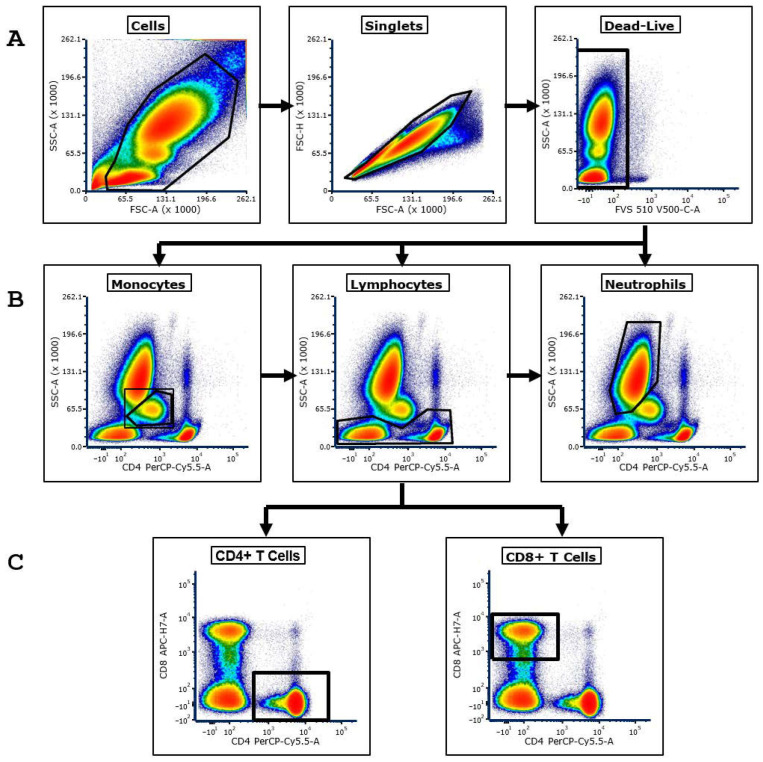
Flow cytometry gating strategy. (**A**) Selection of viable single cells after exclusion of debris, doublets, and dead cells based on forward and side scatter properties and viability staining. (**B**) Identification of major leukocyte populations. (**C**) Identification of CD4^+^ and CD8^+^ T-cell subsets within the lymphocyte gate based on CD4 and CD8 expression.

Flow cytometric acquisition was performed on a BD FACSLyric™ cytometer with BD FACSuite 1.6 Application™ software. Data were analyzed with FCS Express version 7 (De Novo Software, Pasadena, CA, USA). Cells were first gated on SSC-A vs. FSC-A, excluding debris and doublets using FSC-H vs. FSC-A. Viable cells were identified as negative for the viability marker. Lymphocyte, monocyte, and neutrophil populations were manually gated based on FSC-A, SSC-A, and CD4 expression (Figure 1).

CD4^+^ T lymphocytes were stratified by CD25 expression, with positivity thresholds defined by fluorescence-minus-one (FMO) controls applied within each specific cell population. FOXP3-FL and FOXP3-Δ2 expression was assessed within CD4^+^CD25^+^ and CD4^+^CD25^−^ subsets using FMO controls acquired within each respective gate. PD-1 and CTLA-4 positivity thresholds were defined using FMO controls acquired on the total CD4^+^ population and applied uniformly to both CD4^+^CD25^+^ and CD4^+^CD25^−^ subsets. The same FMO-based gating strategy was applied to CD8^+^ T cells, monocytes, and neutrophils using population-specific FMO controls. Positivity thresholds for all markers were established such that fewer than 1% of events in the FMO control fell within the positive region, ensuring stringent and conservative thresholds that minimize false-positive assignment. This criterion was applied consistently across all populations and donors. Representative FMO gates for CD4^+^ subpopulations and for CD8^+^ T cells, monocytes, and neutrophils are shown in Supplementary Figures S1 and S2, respectively.

Data were compiled and analyzed using GraphPad Prism version 10.6.0 (GraphPad Software, San Diego, CA, USA).

### 2.4. Statistics

Statistical analyses were performed using GraphPad Prism version 10.6.0 (GraphPad Software, San Diego, CA, USA) and Python (version 3.11) within the Jupyter Notebook 7.2.2 environment (Miniconda distribution). All comparisons were paired at the donor level. Reported *p*-values were obtained using two-tailed paired Student’s *t*-tests. Given the small sample size, Wilcoxon signed-rank tests were additionally performed as a sensitivity analysis and yielded concordant significance calls across the reported comparisons. A *p*-value < 0.05 was considered statistically significant.

## 3. Results

Peripheral blood samples from six healthy donors were analyzed by multiparametric flow cytometry to describe the basal frequency and distribution of the FOXP3-FL and FOXP3-Δ2 isoforms across leukocyte populations, together with PD-1 and CTLA-4 expression.

### 3.1. General Characterization of Analyzed Populations

A standardized gating strategy (see Figure 1A–C) was used to identify the main leukocyte populations. After eliminating debris, doublets, and dead cells, leukocyte subsets were distinguished using a plot of SSC-A against CD4. This representation allows a clean separation of CD4-high/SSC-low lymphocytes, CD4-low/SSC-intermediate monocytes, and CD4-negative/SSC-high neutrophils. The population with high CD4 levels, which are genuine lymphocytes, served as an internal reference point for defining the boundaries of the lymphocyte gate. Within the lymphocyte compartment, CD4^+^ and CD8^+^ T cells were subsequently identified based on CD4 and CD8 expression.

**Figure 2:**
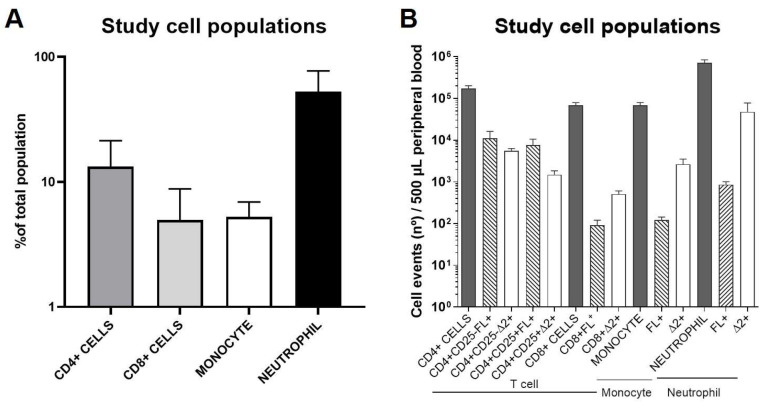
Distribution of circulating leukocyte populations. (**A**) Relative frequency of major leukocyte populations, expressed as percentage of total leukocytes. (**B**) Absolute number of events acquired per population.

Among circulating leukocytes (Figure 2A,B), neutrophils were the dominant population (55.76 ± 19.35%; median 830,470 events [475,698–892,339]). CD4^+^ T cells constituted the next largest compartment (14.59 ± 7.12%), whereas CD8^+^ T cells (5.50 ± 2.03%) and monocytes (5.42 ± 1.34%) were comparatively minor subsets.

### 3.2. Analysis of CD4^+^CD25^−^ T Lymphocytes

**Figure 3:**
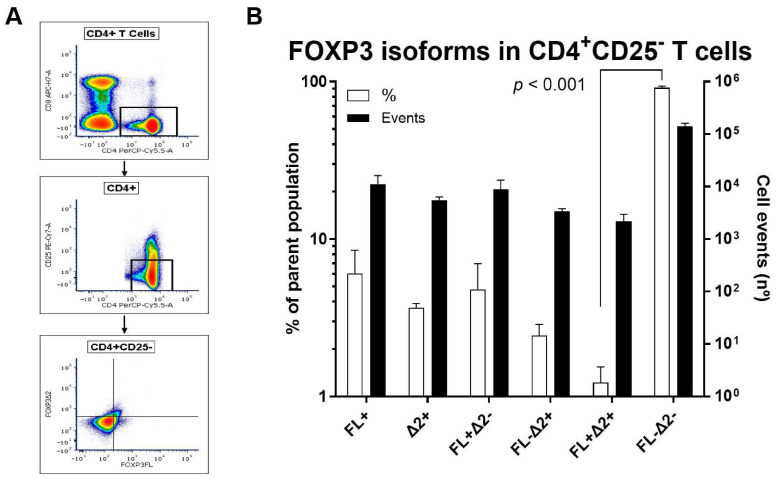
FOXP3 isoform expression in CD4^+^CD25^−^. (**A**) Representative gating strategy for the identification of FOXP3 full-length (FOXP3-FL) and FOXP3-Δ2 isoforms within CD4^+^CD25^−^ T cells. (**B**) Distribution of FOXP3 isoform combinations in CD4^+^CD25^−^ T cells, expressed as percentage of the parent population and as number of acquired events.

Within the CD4^+^CD25^−^ T-cell compartment, most cells lacked detectable FOXP3 expression, with FOXP3-FL^−^FOXP3-Δ2^−^ cells accounting for 91.5 ± 2.1% of the population (Figure 3A,B). Among FOXP3-expressing CD4^+^CD25^−^ T cells, the full-length FOXP3-FL isoform predominated over the Δ2 isoform (6.0 ± 2.5% vs. 3.7 ± 0.24%), with both occurring much less frequently than in CD4^+^CD25^+^ T cells.

Analysis of isoform combinations revealed that FOXP3-FL^+^Δ2^−^ cells were the most prevalent FOXP3^+^ subset, whereas FOXP3-FL^+^Δ2^+^ cells were rare. Table 1 provides a summary of the percentages, absolute numbers of events, and ratios of FL^+^/Δ2^+^ events.

#### PD-1 and CTLA-4 Within CD4^+^CD25^−^ T Cells

**Figure 4:**
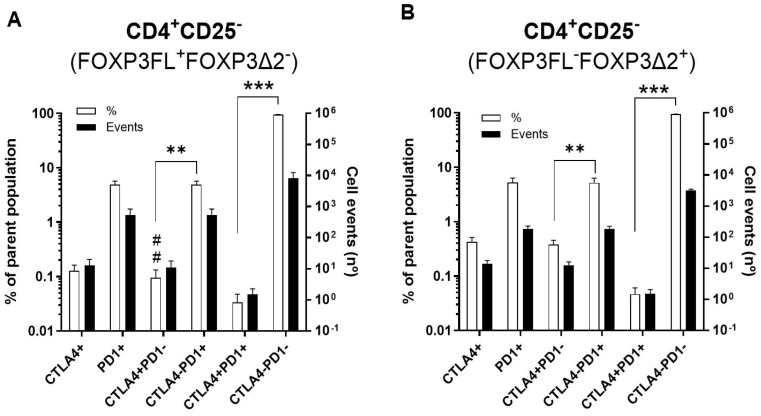
PD-1 and CTLA-4 expression across FOXP3-defined CD4^+^CD25^−^ T-cell subsets. Quantitative analysis of PD-1 and CTLA-4 expression patterns across FOXP3FL+Δ2- (**A**) and FOXP3FL-Δ2+ (**B**) CD4^+^CD25^−^ T-cell subsets, expressed as percentage of the parent population and number of events acquired per 500 µL of peripheral blood. Statistical comparisons were performed using paired two-tailed Student’s *t*-tests. ** *p* < 0.01; *** *p* < 0.001. Asterisks indicate within-panel comparisons between CTLA-4^+^PD-1^−^ and CTLA-4^−^PD-1^+^ cells, or between CTLA-4^+^PD-1^+^ and CTLA-4^−^PD-1^−^ cells, as indicated. ## denotes *p* < 0.01 in direct comparisons between the corresponding checkpoint-defined subsets of FOXP3-FL^+^Δ2^−^ and FOXP3-FL^−^Δ2^+^ cells. Gray arrows indicate values below the detection threshold.

A summary of checkpoint distribution among FOXP3-defined subsets can be found in Figure 4A,B.

Across CD4^+^CD25^−^ T cells, the CTLA-4^−^PD-1^−^ phenotype predominated in all FOXP3-defined subsets, accounting for more than 90% of cells and establishing a checkpoint-negative baseline in this compartment. Among checkpoint-positive cells, PD-1 single-positive cells were more frequent than CTLA-4 single-positive cells.

Direct analysis of the exclusive isoform subsets (FOXP3-FL^+^Δ2^−^ and FOXP3-FL^−^Δ2^+^) showed a similar checkpoint architecture. In both populations, CTLA-4^−^PD-1^+^ cells were significantly more frequent than CTLA-4^+^PD-1^−^ cells, whereas double-negative cells were significantly enriched compared with double-positive cells.

Direct comparison between the two subsets revealed a significantly higher frequency of CTLA-4^+^PD-1^−^ cells in FOXP3-FL^−^Δ2^+^ cells than in FOXP3-FL^+^Δ2^−^ cells, indicating a modest but reproducible enrichment of CTLA-4 single-positive cells in the Δ2-dominant population. Absolute event counts paralleled percentage-based distributions (Table 2). Event ratios further supported these observations: the FL^+^/Δ2^+^ ratio was markedly higher for PD-1^+^ cells (mean ratio = 1.96), indicating a predominance of the FL isoform within the PD-1^+^ subset, whereas CTLA-4^+^ cells showed a ratio below 1 (mean ratio = 0.65), reflecting a relative enrichment of the Δ2 isoform in CTLA-4 single-positive cells.

### 3.3. Analysis of CD4^+^CD25^+^ T Lymphocytes

**Figure 5:**
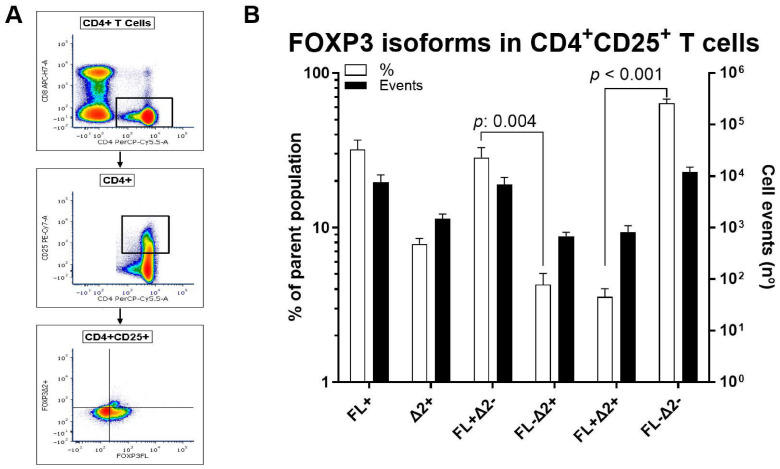
FOXP isoform expression in CD4^+^CD25^+^. (**A**) Representative gating strategy for the identification of FOXP3 full-length (FOXP3-FL) and FOXP3-Δ2 isoforms within CD4^+^CD25^+^ T cells. (**B**) Distribution of FOXP3 isoform combinations in CD4^+^CD25^+^ T cells, expressed as percentage of the parent population and as number of acquired events.

Within the CD4^+^CD25^+^ compartment, the FOXP3-FL^−^Δ2^−^ population represented the largest fraction of cells (63.7 ± 4.1%) (Figure 5A,B). Among FOXP3-expressing cells, the full-length FOXP3-FL isoform predominated (32.0 ± 4.8%), whereas the Δ2 isoform accounted for a smaller fraction (7.8 ± 0.67%). Both isoforms were detected at higher frequencies than in the CD4^+^CD25^−^ compartment.

Analysis of isoform combinations revealed that FOXP3-FL^+^Δ2^−^ cells were the predominant FOXP3^+^ subset (28.4 ± 4.5%), followed by FOXP3-FL^−^Δ2^+^ cells (4.3 ± 0.78%), whereas FOXP3-FL^+^Δ2^+^ cells remained infrequent (3.6 ± 0.45%). FOXP3-FL^+^Δ2^−^ cells were significantly more frequent than FOXP3-FL^−^Δ2^+^ cells, whereas the double-negative population remained significantly more abundant than the double-positive subset.

Detailed percentages, absolute event counts, and FL^+^/Δ2^+^ event ratios are summarized in Table 3. Event ratios confirmed the predominance of the FL isoform in CD4^+^CD25^+^ T cells, with an FL^+^/Δ2^+^ ratio of 4.37. This predominance was even more pronounced when considering exclusive isoform populations, where the FL^+^Δ2^−^/FL^−^Δ2^+^ ratio reached 11.01, indicating that cells expressing only the FL isoform outnumber those expressing only Δ2 by more than 10-fold in this regulatory T-cell compartment.

#### PD-1 and CTLA-4 Within CD4^+^/CD25^+^ T Cells

**Figure 6:**
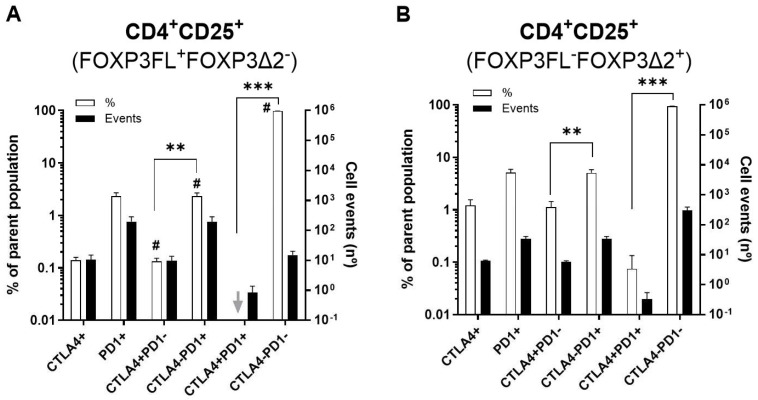
PD-1 and CTLA-4 expression across FOXP3FL+Δ2- -defined CD4+CD25− T-cell subsets (**A**) and FOXP3FL-Δ2+ (**B**) CD4+CD25− T-cell subsets. Quantitative analysis of PD-1 and CTLA-4 expression patterns across FOXP3-defined CD4^+^CD25^+^ T-cell subsets, expressed as percentage of the parent population and number of acquired events per 500 µL of peripheral blood. Statistical comparisons were performed using paired two-tailed Student’s *t*-tests. ** *p* < 0.01; *** *p* < 0.001. Asterisks indicate within-panel comparisons between CTLA-4^+^PD-1^−^ and CTLA-4^−^PD-1^+^ cells, or between CTLA-4^+^PD-1^+^ and CTLA-4^−^PD-1^−^ cells, as indicated. # denotes direct comparison between the corresponding checkpoint-defined subsets of FOXP3-FL^+^Δ2^−^ and FOXP3-FL^−^Δ2^+^ cells. # *p* < 0.05. Gray arrows indicate values below the detection threshold.

Checkpoint distribution across FOXP3-defined subsets is summarized in Figure 6.

Across FOXP3-defined CD4^+^CD25^+^ subsets, the CTLA-4^−^PD-1^−^ phenotype predominated, accounting for more than 90% of cells in every group. Among checkpoint-positive cells, PD-1 single-positive cells were consistently more frequent than CTLA-4 single-positive cells.

Direct analysis of the exclusive isoform subsets (FOXP3-FL^+^Δ2^−^ and FOXP3-FL^−^Δ2^+^) showed a comparable checkpoint architecture. In both populations, CTLA-4^−^PD-1^+^ cells were significantly more frequent than CTLA-4^+^PD-1^−^ cells, whereas double-negative cells were significantly enriched compared with double-positive cells.

Direct comparison between the two subsets revealed significantly higher frequencies of CTLA-4^+^PD-1^−^ and CTLA-4^−^PD-1^+^ cells in FOXP3-FL^−^Δ2^+^ cells than in FOXP3-FL^+^Δ2^−^ cells. CTLA-4-expressing subsets remained rare across all FOXP3-defined populations. Absolute event counts mirrored percentage-based distributions (Table 4). Event ratios confirmed a preferential association of PD-1 with the FL isoform and CTLA-4 with the Δ2 isoform within CD4^+^CD25^+^ T cells.

### 3.4. Analysis of CD8^+^ T Lymphocytes

**Figure 7:**
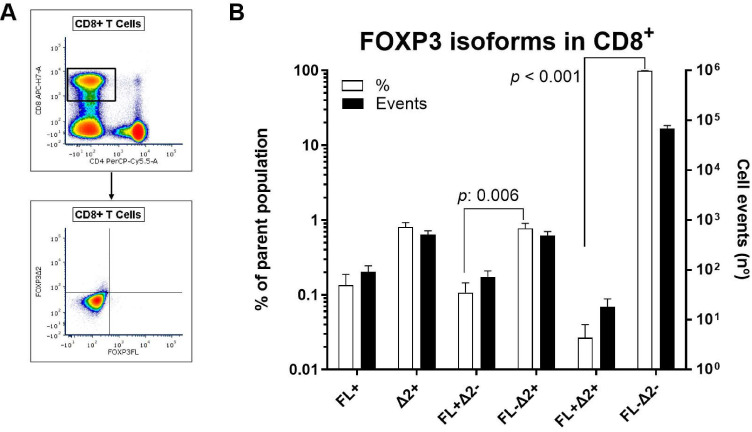
FOXP isoform expression in CD8^+^. (**A**) Representative gating strategy for the identification of FOXP3 full-length (FOXP3-FL) and FOXP3-Δ2 isoforms within CD8^+^ T cells. (**B**) Distribution of FOXP3 isoform combinations in CD8^+^ T cells, expressed as percentage of the parent population and as number of acquired events.

Within CD8^+^ T lymphocytes, the FOXP3-FL^−^Δ2^−^ population predominated (99.1 ± 0.11%), indicating that the vast majority of CD8^+^ T cells lacked detectable FOXP3 expression (Figure 7A,B). Among FOXP3-expressing CD8^+^ cells, the Δ2 isoform was detected more frequently than the full-length FOXP3-FL isoform (0.80 ± 0.12% vs. 0.14 ± 0.04%, respectively; *p* = 0.006). This contrasted with CD4^+^ T cells, in which the full-length isoform predominated.

Analysis of isoform combinations revealed that FOXP3-FL^−^Δ2^+^ cells represented the predominant FOXP3^+^ subset (0.78 ± 0.13%), followed by FOXP3-FL^+^Δ2^−^ cells (0.11 ± 0.04%) (*p* = 0.006), whereas double-positive FOXP3-FL^+^Δ2^+^ cells were rare (0.03 ± 0.01%). The double-negative population remained significantly more abundant than the double-positive subset (*p* < 0.001).

Detailed percentages, absolute event counts, and FL^+^/Δ2^+^ event ratios are summarized in Table 5. Event ratios confirmed the predominance of the Δ2 isoform in CD8^+^ T cells, with an FL^+^/Δ2^+^ ratio of 0.20, which was further accentuated when considering exclusive isoform populations (FL^+^Δ2^−^/FL^−^Δ2^+^ = 0.18).

#### PD-1 and CTLA-4 Expression Within CD8^+^ T Cells

**Figure 8:**
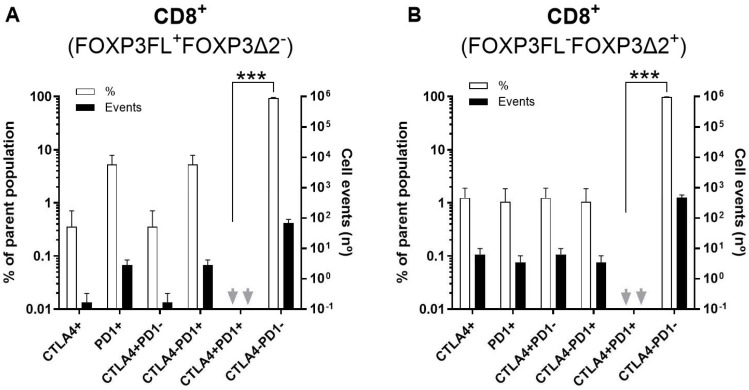
PD-1 and CTLA-4 expression across FOXP3-defined CD8^+^ T-cell subsets. (**A**) Quantitative analysis of PD-1 and CTLA-4 expression patterns across FOXP3-defined CD8^+^ T-cell subsets, expressed as percentage of the parent population and number of acquired events per 500 µL of peripheral blood. (**B**) Representative flow cytometry plots showing PD-1 and CTLA-4 expression in FOXP3-defined CD8^+^ T-cell subsets. Statistical comparisons were performed using paired two-tailed Student’s *t*-tests. *** *p* < 0.001. Asterisks indicate within-panel comparisons between CTLA-4^+^PD-1^−^ and CTLA-4^−^PD-1^+^ cells, or between CTLA-4^+^PD-1^+^ and CTLA-4^−^PD-1^−^ cells, as indicated. Gray arrows indicate values below the detection threshold.

PD-1 and CTLA-4 expression were evaluated in the predominant isoform-defined CD8^+^ T-cell subsets (FOXP3-FL^+^Δ2^−^ and FOXP3-FL^−^Δ2^+^) (Figure 8A,B). Because FOXP3 expression in CD8^+^ T cells was extremely rare, only descriptive analyses restricted to the exclusive FOXP3-FL^+^Δ2^−^ and FOXP3-FL^−^Δ2^+^ subsets were performed.

In both subsets, the CTLA-4^−^PD-1^−^ phenotype predominated, accounting for more than 94% of cells. Among checkpoint-positive cells, PD-1 single-positive cells were slightly more frequent than CTLA-4 single-positive cells, whereas CTLA-4^+^PD-1^+^ double-positive events were not detected in any donor. These observations indicate that checkpoint expression in CD8^+^ T cells was minimal and largely restricted to PD-1 single-positive cells.

Detailed percentages, absolute event counts, and FL^+^/Δ2^+^ event ratios are summarized in Table 6. Event ratios reflected the overall Δ2 predominance in CD8^+^ T cells (FL^+^/Δ2^+^ = 0.20), and within checkpoint-positive subsets, CTLA-4^+^ cells showed a higher Δ2 enrichment (Δ2^+^/FL^+^ = 5.61) compared to PD-1^+^ cells (FL^+^/Δ2^+^ = 0.76).

### 3.5. Analysis of Monocytes

**Figure 9:**
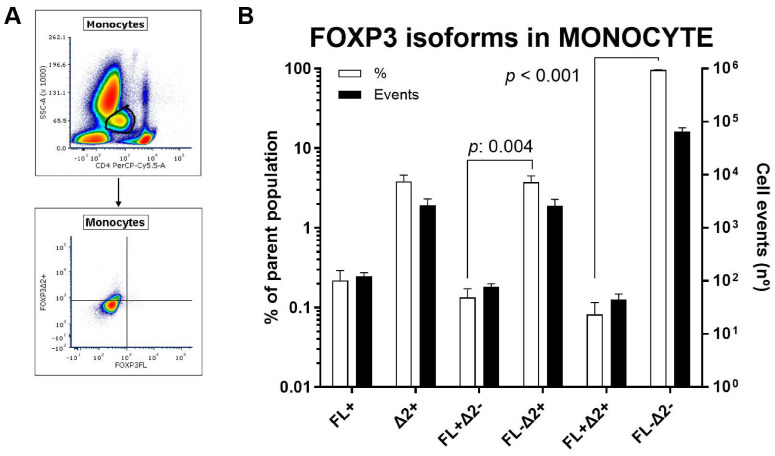
FOXP3 isoform expression in monocytes. (**A**) Representative gating strategy for the identification of FOXP3 full-length (FOXP3-FL) and FOXP3-Δ2 isoforms within monocytes. (**B**) Distribution of FOXP3 isoform combinations in monocytes, expressed as percentage of the parent population and as number of acquired events.

Monocytes were predominantly FOXP3-FL^−^Δ2^−^ (96.1 ± 0.78%), indicating that FOXP3 expression was detected only in a small fraction of cells (Figure 9A,B). Among FOXP3-expressing monocytes, the Δ2 isoform predominated (3.8 ± 0.77%; median 1909 [1765–2389] events per 500 µL), whereas the full-length FOXP3-FL isoform accounted for a much smaller fraction (0.22 ± 0.07%; median 120 [84–142] events). This distribution resembled that observed in CD8^+^ T cells and contrasted with the pattern in CD4^+^ T cells, in which the full-length isoform predominated.

Analysis of isoform combinations showed that FOXP3-FL^−^Δ2^+^ cells represented the predominant FOXP3^+^ subset (3.7 ± 0.76%), followed by FOXP3-FL^+^Δ2^−^ cells (0.13 ± 0.04%), whereas FOXP3-FL^+^Δ2^+^ cells remained rare (0.08 ± 0.08%). FOXP3-FL^−^Δ2^+^ cells were significantly more frequent than FOXP3-FL^+^Δ2^−^ cells (*p* = 0.004), and the double-negative population remained significantly more abundant than the double-positive subset (*p* < 0.001).

Detailed percentages, absolute event counts, and FL^+^/Δ2^+^ event ratios are summarized in Table 7. Event ratios confirmed the marked predominance of the Δ2 isoform in monocytes (FL^+^/Δ2^+^ = 0.05), the lowest ratio observed across all cell populations studied.

#### PD-1 and CTLA-4 Expression Within Monocytes

**Figure 10:**
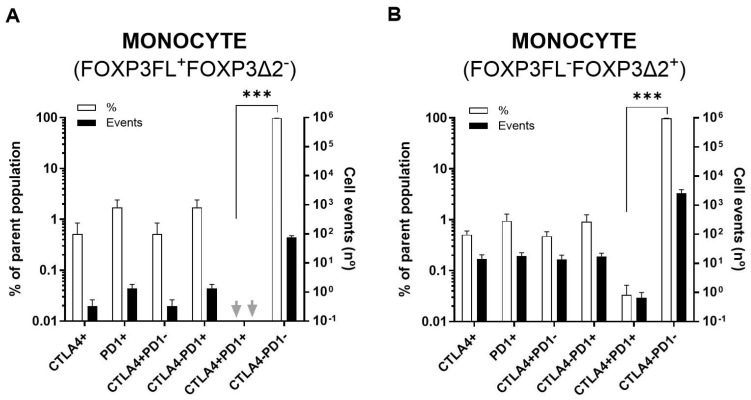
PD-1 and CTLA-4 expression across FOXP3-defined monocytes. (**A**) Quantitative analysis of PD-1 and CTLA-4 expression patterns across FOXP3-defined monocyte subsets, expressed as percentage of the parent population and number of acquired events per 500 µL of peripheral blood. (**B**) Representative flow cytometry plots showing PD-1 and CTLA-4 expression in FOXP3-defined monocyte subsets. Statistical comparisons were performed using paired two-tailed Student’s *t*-tests. *** *p* < 0.001. Asterisks indicate within-panel comparisons between CTLA-4^+^PD-1^−^ and CTLA-4^−^PD-1^+^ cells, or between CTLA-4^+^PD-1^+^ and CTLA-4^−^PD-1^−^ cells, as indicated. Gray arrows indicate values below the detection threshold.

To compare checkpoint expression according to FOXP3 isoform identity, analyses were restricted to the exclusive FOXP3-FL^+^Δ2^−^ and FOXP3-FL^−^Δ2^+^ subsets. FOXP3-FL^+^Δ2^+^ cells were too infrequent for reliable analysis. (Figure 10A,B).

In both subsets, the CTLA-4^−^PD-1^−^ phenotype predominated, accounting for more than 98% of cells. Among checkpoint-positive cells, PD-1 single-positive cells were slightly more frequent than CTLA-4 single-positive cells, whereas CTLA-4^+^PD-1^+^ events were extremely rare or absent. In FOXP3-FL^+^Δ2^−^ monocytes, PD-1 single-positive cells represented the most frequent checkpoint-positive subset, whereas CTLA-4 single-positive cells were detected at slightly lower frequencies. A similar pattern was observed in FOXP3-FL^−^Δ2^+^ monocytes, although overall checkpoint expression remained extremely low.

Overall, both PD-1 and CTLA-4 were detected at minimal frequencies in monocytes, with PD-1 expression slightly exceeding CTLA-4 (Table 8). Event ratios within checkpoint-positive subsets were consistent with the overall Δ2 predominance in monocytes, with both CTLA-4^+^ (Δ2^+^/FL^+^ = 17.83) and PD-1^+^ cells (Δ2^+^/FL^+^ = 9.63) showing markedly higher Δ2 event counts, reflecting the global isoform distribution rather than a specific checkpoint-isoform association.

### 3.6. Analysis of Neutrophils

**Figure 11:**
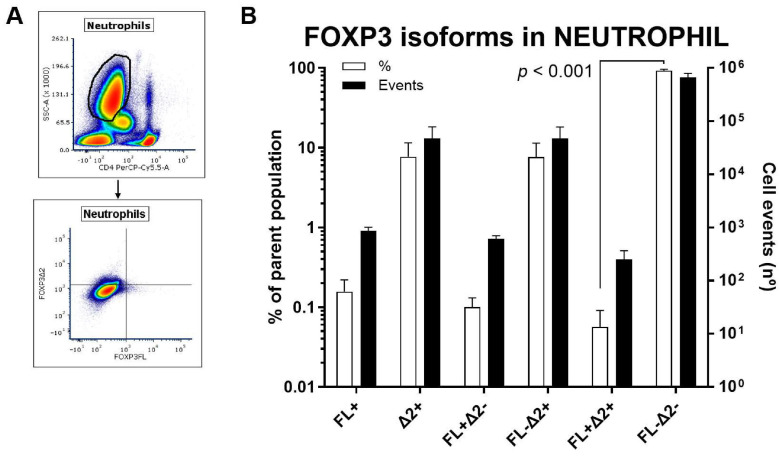
FOXP isoform expression in neutrophils. (**A**) Representative gating strategy for the identification of FOXP3 full-length (FOXP3-FL) and FOXP3-Δ2 isoforms within neutrophils. (**B**) Distribution of FOXP3 isoform combinations in neutrophils, expressed as percentage of the parent population and as number of acquired events.

Neutrophils were predominantly FOXP3-FL^−^Δ2^−^ (92.2 ± 3.83%), indicating that most cells lacked detectable FOXP3 expression (Figure 11A,B). Among FOXP3-expressing neutrophils, the Δ2 isoform was detected more frequently (7.7 ± 3.82%; median 16,149 [7582–39,890] events).

Analysis of isoform combinations showed that FOXP3-FL^−^Δ2^+^ cells represented the predominant FOXP3^+^ subset (7.6 ± 3.8%), followed by FOXP3-FL^+^Δ2^−^ cells (0.10 ± 0.03%), whereas double-positive FOXP3-FL^+^Δ2^+^ cells remained rare (0.06 ± 0.03%). Overall, Δ2 expression was more frequently detected than the full-length isoform in neutrophils, although FOXP3 expression in this population remained markedly lower than in CD4^+^ T cells.

Detailed percentages, absolute event counts, and FL^+^/Δ2^+^ event ratios are summarized in Table 9. The FL^+^/Δ2^+^ event ratio in neutrophils was 0.07 (mean of donor-specific ratios), indicating a pronounced predominance of FOXP3-Δ2 over FOXP3-FL in this compartment. These data demonstrate that FOXP3 expression in neutrophils is almost exclusively accounted for by the Δ2 isoform.

#### PD-1 and CTLA-4 Expression Within Neutrophils

**Figure 12:**
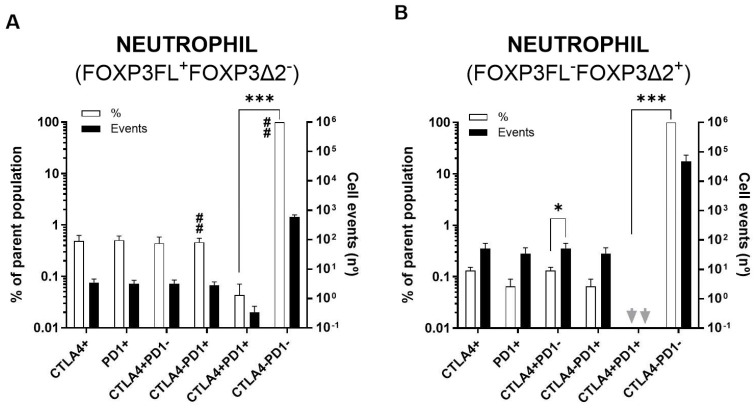
PD-1 and CTLA-4 expression across FOXP3-defined neutrophil subsets (**A**) Quantitative analysis of PD-1 and CTLA-4 expression patterns across FOXP3-defined neutrophil subsets, expressed as percentage of the parent population and number of acquired events per 500 µL of peripheral blood. (**B**) Representative flow cytometry plots showing PD-1 and CTLA-4 expression in FOXP3-defined neutrophil subsets. Statistical comparisons were performed using paired two-tailed Student’s *t*-tests. * *p* < 0.05; *** *p* < 0.001. Asterisks indicate within-panel comparisons between CTLA-4^+^PD-1^−^ and CTLA-4^−^PD-1^+^ cells, or between CTLA-4^+^PD-1^+^ and CTLA-4^−^PD-1^−^ cells, as indicated. ## *p* < 0.01 with direct comparison between the corresponding checkpoint-defined subsets of FOXP3-FL^+^Δ2^−^ and FOXP3-FL^−^Δ2^+^ cells. Gray arrows indicate values below the detection threshold.

To compare checkpoint expression according to FOXP3 isoform identity, analyses were restricted to the exclusive FOXP3-FL^+^Δ2^−^ and FOXP3-FL^−^Δ2^+^ subsets. FOXP3-FL^+^Δ2^+^ cells were too infrequent for reliable analysis (Figure 12A,B).

In both subsets, the CTLA-4^−^PD-1^−^ phenotype predominated, accounting for more than 99% of cells. Checkpoint-positive events were exceedingly rare, with no evidence of distinct CTLA-4- or PD-1-expressing subsets. Within the FOXP3-FL^+^Δ2^−^ neutrophil population, CTLA-4 single-positive and PD-1 single-positive cells were detected only at very low frequencies and showed comparable representation. A similar pattern was observed in FOXP3-FL^−^Δ2^+^ neutrophils, where CTLA-4 and PD-1 expression were detected only at minimal frequencies, although the number of events was higher. Overall, both checkpoint molecules were expressed at extremely low levels in neutrophils.

Detailed percentages, absolute event counts, and FL/Δ2 ratios are summarized in Table 10. Event ratios within checkpoint-positive subsets were consistent with the overall Δ2 predominance in neutrophils, with both CTLA-4^+^ (Δ2^+^/FL^+^ = 10.68) and PD-1^+^ cells (Δ2^+^/FL^+^ = 7.36) showing markedly higher Δ2 event counts, reflecting the global isoform distribution rather than a specific checkpoint-isoform association.

### 3.7. FOXP3 Isoform Balance Is Differentially Modulated Across Immune Cell Types and Checkpoint-Defined States

**Figure 13:**
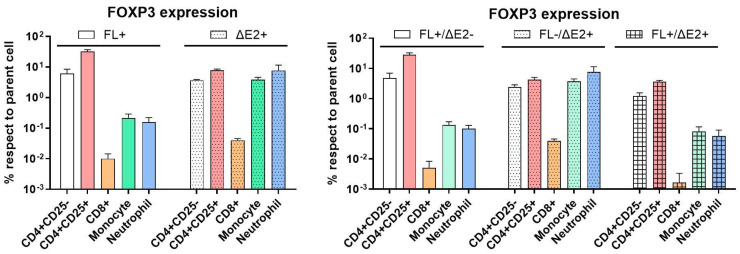
FOXP3 isoform expression across immune cell subsets. FOXP3 Full-Length and ΔE2 variants across major immune cell populations. White: CD4+CD25- T cells; pink: CD4+CD25+ T cells; orange: CD8+ T cells; Green: monocyte; Blue: neutrophil.

**Figure 14:**
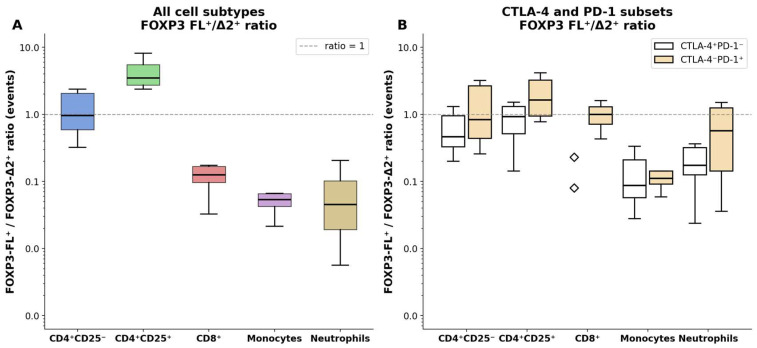
FOXP3 isoform balance across immune cell subsets and CTLA-4/PD-1-defined functional states. (**A**) FOXP3-FL^+^/FOXP3-Δ2^+^ ratios across major immune cell populations. (**B**) FOXP3-FL^+^/FOXP3-Δ2^+^ ratios within CTLA-4/PD-1-defined subsets. Each data point represents an individual donor (*n* = 6). Boxplots display the median, interquartile range, and whiskers (minimum to maximum values). The dashed line indicates a ratio of 1. Blue: CD4+CD25- T cells; green: CD4+CD25+ T cells; orange: CD8+ T cells; purple: monocyte; brown: neutrophil.

To assess the relative predominance of FOXP3 isoforms across immune cell populations, we represented the percentage of cells in each PBMC subset that expressed each isoform (Figure 13). The full-length FOXP3 isoform predominated in CD4^+^CD25^+^ T cells, whereas FOXP3-Δ2 was relatively enriched in CD8^+^ T cells and innate immune populations. We also calculated the FOXP3-FL^+^/FOXP3-Δ2^+^ ratio (Figure 14A) of events to visualize the contrast among the different cell subtypes. In CD4^+^CD25^+^ regulatory T cells, the full-length FOXP3 isoform predominated. In contrast, FOXP3-Δ2 was relatively enriched in CD8^+^ T cells, monocytes, and neutrophils. These findings suggest that cell lineage may influence the relative expression of FOXP3 isoforms.

The FOXP3-FL^+^/FOXP3-Δ2^+^ ratio further varied according to CTLA-4/PD-1-defined functional states (Figure 14B), particularly within CD4^+^ T-cell compartments. Notably, CTLA4^−^PD1^+^ subsets exhibited higher FOXP3-FL/Δ2 ratios compared to CTLA4^+^PD1^−^ cells, indicating a relative enrichment of the full-length FOXP3 isoform in PD-1-expressing populations. This pattern was most pronounced in CD4^+^CD25^+^ T cells, whereas CD8^+^ T cells displayed a more balanced distribution. In contrast, innate immune populations showed consistently lower ratios without a clear association with checkpoint-defined states.

## 4. Discussion

In this study, we systematically characterized for the first time the distribution of FOXP3 isoforms across human peripheral blood immune cell populations and examined their association with CTLA-4 and PD-1 expression. Although FOXP3 expression has been extensively characterized in regulatory T cells (Tregs), its distribution across the broader hematopoietic compartment remains poorly defined.

The principal finding of this study is that FOXP3-FL predominates in CD4+CD25+ T cells, whereas FOXP3-Δ2 is preferentially detected in other peripheral immune cell populations, including CD8+ T cells, monocytes, and neutrophils, although at low frequencies. In particular, we show that: (a) FOXP3 isoform balance varies markedly across immune cell populations; (b) the highest FOXP3 expression was observed in CD4^+^CD25^+^ T cells (canonical Treg cells); (c) FOXP3-FL was detected in approximately 30% of the CD4+CD25+ T-cell population; and (d) the full-length isoform (FOXP3-FL) predominated over FOXP3-Δ2, with a FL^+^/Δ2^+^ ratio above three.

Although some studies have reported that the vast majority (>90%) of CD4 + CD25^high^ T cells are FOXP3+ [28], analyses performed on the entire CD4 + CD25+ T-cell compartment, regardless of CD25 expression levels, have shown FOXP3 positivity ranging from 27% to 52.7% [29]. These findings are consistent with our results.

In CD4^+^CD25^−^ T cells, both isoforms were detected at considerably lower frequencies, with a FOXP3-FL^+^/FOXP3-Δ2^+^ ratio approaching unity, indicating a more balanced isoform distribution in this non-regulatory compartment. In contrast, CD8^+^ T cells, monocytes, and neutrophils displayed markedly reduced overall FOXP3 expression, and when present, FOXP3 was predominantly represented by the Δ2 isoform. Despite the low absolute frequencies, the consistent predominance of FOXP3-Δ2 across these lineages suggests that isoform distribution is associated with cell lineage rather than occurring stochastically. However, it must be taken into account that the technical detection sensitivity could play a role in the low overall expression detected.

The simultaneous detection of different isoforms within the same cell may help improve the prediction of the final cellular response. In this context, we analyzed isoform ratios as a unified predictive parameter that we speculate could be useful both in autoimmune disease scenarios and in therapy-induced immune responses.

Since the initial description of full-length (FOXP3-FL) and exon-2-deficient (FOXP3-Δ2) isoforms in humans by Allan et al. [4], several studies have attempted to define their relative expression and functional roles. Aarts-Riemens et al. [22] demonstrated that both isoforms can be co-expressed in human T cells following FOXP3 transduction, without a fixed proportional relationship between them. In contrast, Mailer et al. [14] reported that FOXP3-Δ2 transcripts may exceed FOXP3-FL at the transcriptional level, highlighting that mRNA abundance does not necessarily reflect protein expression.

Importantly, most available data on FOXP3 isoforms in human peripheral blood derive from mRNA-based analyses. For example, Kristensen et al. [30] reported that FOXP3-Δ2 accounts for approximately 30% of total FOXP3 transcripts in CD4^+^ T cells from healthy donors, with this proportion increasing in autoimmune conditions such as Hashimoto’s thyroiditis and Graves’ disease. Similarly, Zhdanov et al. [31] described comparable levels of FOXP3 isoforms at the transcriptional level, with a relative predominance of the full-length variant.

In contrast, studies assessing FOXP3 isoforms at the protein level are scarce. Notably, Free et al. [32], using flow cytometry, observed that FOXP3-FL-expressing cells predominate over FOXP3-Δ2^+^ cells in peripheral blood Tregs from healthy donors, supporting a physiological enrichment of the full-length isoform.

Taken together, these findings suggest that while transcriptional studies provide valuable insight into FOXP3 splicing, they may not accurately reflect isoform distribution at the protein level. In this context, our results (Figure 14) extend previous observations by demonstrating, at the protein level, that FOXP3-FL predominates within regulatory T cells, while a relative enrichment of FOXP3-Δ2 is observed in non-regulatory immune populations. However, to our knowledge, the distribution of FOXP3 isoforms across non-CD4^+^ immune cell lineages has not been systematically investigated [33,34], underscoring the novelty of our approach. Although FOXP3-Δ2 has been proposed to contribute to the regulation and functional balance of human Tregs [10], the biological significance of its expression in non-T-cell populations remains poorly understood.

We next examined the relationship between FOXP3 isoforms and immune checkpoint expression. In both CD4^+^CD25^−^ and CD4^+^CD25^+^ T-cell populations, PD-1 expression consistently exceeded that of CTLA-4, regardless of FOXP3 isoform. Notably, within these compartments, FOXP3-Δ2-expressing cells exhibited higher PD-1 expression compared to FOXP3-FL-expressing cells.

Data addressing the relationship between FOXP3 isoforms and checkpoint expression are scarce. However, Sambucci et al. [35] reported increased PD-1 expression in memory Treg cells from patients with multiple sclerosis, a context of chronic immune activation, suggesting that dysfunctional or persistently stimulated regulatory populations may upregulate inhibitory checkpoints.

In this context, and considering that FOXP3-Δ2 has been associated with reduced regulatory stability and function [36], the increased PD-1 expression observed in FOXP3-Δ2^+^ cells in our study could be consistent with a compensatory mechanism to limit activation in a potentially less stable regulatory subset.

One of the most intriguing observations of the present study is the detection of FOXP3 expression in leukocyte populations beyond the conventional CD4 + CD25+ regulatory T-cell compartment. Outside the CD4^+^ compartment, FOXP3^+^ cells were detected at low frequencies, and checkpoint expression was generally limited. In monocytes, a consistent trend toward higher PD-1 compared to CTLA-4 was observed across both FOXP3 isoforms. In contrast, neutrophils showed relatively higher CTLA-4 expression than PD-1 for both isoforms, while in CD8^+^ T cells, this pattern was only observed within the FOXP3-Δ2-expressing subset. Although FOXP3 is widely recognized as the defining transcription factor of Tregs, increasing evidence suggests that its expression is not strictly restricted to these cells.

FOXP3 expression has been reported in activated CD8+ T cells, B lymphocytes, NK cells, and monocyte-derived populations, although its expression levels are generally lower and often more transient than those observed in classical Tregs. The biological significance of this expression remains incompletely understood. One possibility is that FOXP3 participates in activation-induced feedback mechanisms that limit excessive immune responses. Alternatively, FOXP3 expression in non-CD4+ populations may reflect context-dependent regulatory programs that become relevant under inflammatory or pathological conditions.

The relatively low expression levels observed in some leukocyte subsets raise the possibility that FOXP3 may exert subtle regulatory effects that are highly dependent on cellular context, cofactor availability, and signaling networks. Therefore, the presence of FOXP3 transcripts should not necessarily be interpreted as evidence of a fully developed regulatory phenotype but rather as an indication of potential participation in broader immune regulatory mechanisms. Further functional studies will be required to determine whether the detected FOXP3 isoforms contribute directly to cellular behavior in these populations.

In addition, the relative balance between FOXP3 isoforms, expressed as the FOXP3-FL^+^/FOXP3-Δ2^+^ ratio, may provide further insight into the functional state of immune cell subsets. In our study, higher ratios were observed in regulatory CD4^+^CD25^+^ T cells, whereas lower ratios characterized CD8^+^ T cells and innate immune populations. Notably, although all cell populations followed a consistent isoform distribution pattern for both CTLA-4^+^ and PD-1^+^ subsets, PD-1^+^ cells were systematically associated with a higher FL^+^/Δ2^+^ ratio compared to their CTLA-4^+^ counterparts across all lineages examined, suggesting that PD-1 expression is more closely linked to the FOXP3-FL isoform. Experimental studies have suggested that the co-expression of FOXP3 isoforms may be required for optimal regulatory T-cell function [37]. It should be noted, however, that our analyses were performed under basal conditions in healthy individuals and that FOXP3 isoform balance may differ in states of immune activation or dysregulation. In this context, a relative increase in FOXP3-Δ2 expression could be associated with reduced regulatory stability or a more plastic phenotype. Although these interpretations remain speculative, changes in the FOXP3-FL^+^/FOXP3-Δ2^+^ ratio may have potential value as indicators of immune status and warrant further investigation in states of immune activation and in pathological settings.

Beyond the descriptive characterization presented here, our findings may have important implications for understanding immune regulation in pathological conditions. Regulatory T cells are highly enriched in many tumors and constitute a major mechanism of immune suppression within the tumor microenvironment. Their suppressive activity is closely associated with the expression of immune checkpoint molecules, including CTLA-4, PD-1, TIGIT, and LAG-3, which are currently targeted by several immunotherapeutic strategies.

Accumulating evidence indicates that human FOXP3 isoforms exhibit distinct transcriptional activities and may differentially influence the expression of genes involved in regulatory function. Although the present study was not designed to assess checkpoint expression directly, the differential distribution of FOXP3 isoforms observed among immune cell subsets raises the possibility that specific isoform patterns may contribute to heterogeneity in checkpoint regulation and suppressive capacity. Such mechanisms could be particularly relevant in cancer, where alterations in Treg frequency.

This study establishes a baseline framework for the characterization of FOXP3 isoform distribution across peripheral blood mononuclear cell (PBMC) subsets in healthy individuals and provides a cytometry-based approach for the simultaneous evaluation of FOXP3-FL and FOXP3-Δ2 expression. The relatively small cohort of healthy donors and the low frequency of FOXP3^+^ events, particularly outside the CD4^+^ compartment, highlight the importance of expanding these analyses in larger and more diverse populations to further refine and validate the observed expression patterns. A key objective of this work was to map the distribution of FOXP3 isoforms across PBMC populations and to explore potential coordinated patterns of expression that may reflect underlying regulatory imbalances. In this context, future studies assessing FOXP3 isoforms under conditions of immune activation or disease will be instrumental in elucidating their biological and translational relevance, particularly in non-CD4^+^ immune cell subsets where their functional significance remains to be fully defined. Since Foxp3 isoforms exert pleiotropic effects on different factors and can modulate the microenvironment, the FL/Δ2 ratio calculation could be a more valuable predictive parameter; this must be validated in further studies with immune activation, though.

In addition, integrating isoform-level protein expression with transcriptomic datasets, including single-cell RNA sequencing approaches, represents a promising avenue to further resolve cell-type-specific FOXP3 expression programs. Although such analyses were beyond the scope of the present study and no RNA-seq data were available, future multi-omics integration may provide deeper mechanistic insight into FOXP3 regulation across immune compartments. The influence of biological variables such as sex on FOXP3 expression patterns also represents an important area for future investigation. While current evidence remains heterogeneous and sometimes conflicting [38,39], incorporating such parameters in larger cohorts may help clarify their contribution to inter-individual variability.

Finally, the associations observed between FOXP3 isoforms and checkpoint molecule expression should be interpreted as descriptive findings that open new hypotheses regarding low-level regulatory programs outside the conventional Treg compartment. Future functional studies will be essential to determine the biological significance of these observations and to establish whether they reflect active regulatory mechanisms or context-dependent expression signatures.

## 5. Conclusions

In conclusion, this study demonstrates that FOXP3 isoform distribution is strongly associated with immune cell lineage and reveals distinct patterns of FOXP3-FL and FOXP3-Δ2 expression across peripheral blood immune populations at the protein level. Regulatory CD4^+^CD25^+^ T cells showed a clear predominance of the full-length isoform, supporting its association with stable regulatory function, whereas non-regulatory populations, including CD8^+^ T cells, monocytes, and neutrophils, displayed a relative enrichment of FOXP3-Δ2. Furthermore, the differential association of FOXP3 isoforms with PD-1 and CTLA-4 expression suggests that FOXP3 splicing may be linked to distinct immunoregulatory states, with FOXP3-Δ2 potentially reflecting a less stable or more plastic phenotype. Although the biological significance of FOXP3 expression outside the CD4^+^ compartment remains to be clarified, these findings provide novel evidence that the FOXP3-FL^+^/FOXP3-Δ2^+^ balance could represent a useful indicator of immune status. Overall, this work expands current understanding of FOXP3 isoform biology in human immune cells and highlights the need for further functional studies under conditions of immune activation and disease.

## Figures and Tables

**Table 1:** FOXP3 isoform distribution in CD4+CD25^−^ T cells. Mean ± SEM percentages and absolute event counts of FOXP3-FL and FOXP3-Δ2 isoform combinations within CD4^+^CD25^−^ T cells. FL^+^/Δ2^+^ and Δ2^+^/FL^+^ event ratios were calculated for each donor individually and are presented as the mean of donor-specific ratios. FL: full-length Foxp3 isoform; Δ2: exon-2-lacking Foxp3 isoform; SEM: standard error of the mean.

	%		Events		Events Ratio (Mean)					
CD4^+^CD25^−^	Mean	SEM	Mean	SEM	FL+/Δ2+	Δ2+/FL+	FL+Δ2-/ FL-Δ2+	FL^−^Δ2^+^/ FL^+^Δ2^−^	FL^+^Δ2^+^/ FL^−^Δ2^−^	FL^−^Δ2^−^/ FL^+^Δ2^+^
FOXP3-FL^+^	6.02	2.47	11,095.00	5082.07	1.73					
FOXP3-Δ2^+^	3.66	0.24	5540.00	777.07		1.30				
FOXP3-FL^+^Δ2^−^	4.79	2.17	8914.50	4359.54			3.93			
FOXP3-FL^−^Δ2^+^	2.44	0.43	3359.50	408.54				1.54		
FOXP3-FL^+^Δ2^+^	1.23	0.32	2180.50	767.08					0.01	
FOXP3-FL^−^Δ2^−^	91.51	2.10	139,908.83	20,220.20						110.72

**Table 2:** PD-1 and CTLA-4 expression across FOXP3 isoform-defined CD4^+^CD25^−^ T-cell subsets. Mean ± SEM percentages and absolute event counts of CTLA-4/PD-1 expression patterns within FOXP3-FL^+^ and FOXP3-Δ2^+^ CD4^+^CD25^−^ T cells. FL^+^/Δ2^+^ and Δ2^+^/FL^+^ event ratios were calculated for each donor individually and are presented as the mean of donor-specific ratios. FL: full-length Foxp3 isoform; Δ2: exon-2-lacking Foxp3 isoform; SEM: standard error of the mean.

	(FOXP3-FL^+^)		(FOXP3-Δ2^+^)		(FOXP3-FL^+^)		(FOXP3-Δ2^+^)		EVENTS RATIO	
Subpopulation (CD4^+^CD25^−^)	% (Mean)	SEM	% (Mean)	SEM	Events (Mean)	SEM	Events (Mean)	SEM	FL^+^/Δ2^+^	Δ2^+^/FL^+^
CTLA-4^+^	0.32	0.10	0.66	0.23	42.17	24.95	43.67	20.92	0.65	2.01
PD-1^+^	5.67	0.76	6.14	0.95	690.67	334.08	334.33	61.32	1.96	1.61
CTLA-4^+^PD-1^−^	0.27	0.08	0.56	0.18	35.00	20.33	36.50	16.28	0.64	2.40
CTLA-4^−^PD-1^+^	5.61	0.75	6.04	0.95	683.50	330.36	327.17	58.08	2.01	1.61
CTLA-4^+^PD-1^+^	0.05	0.02	0.10	0.05	7.17	4.87	7.17	4.71	0.78	0.96
CTLA-4^−^PD-1^−^	93.98	0.82	93.25	0.94	10,364.67	4749.54	5165.83	721.08	1.73	1.28

**Table 3:** FOXP3 isoform distribution in CD4^+^CD25^+^ T cells. Mean ± SEM percentages and absolute event counts of FOXP3-FL and FOXP3-Δ2 isoform combinations within CD4^+^CD25^+^ T cells. FL^+^/Δ2^+^ and Δ2^+^/FL^+^ event ratios were calculated for each donor individually and are presented as the mean of donor-specific ratios. FL: full-length Foxp3 isoform; Δ2: exon-2-lacking Foxp3 isoform; SEM: standard error of the mean.

	%		Events		Events Ratio (Mean)					
CD4^+^CD25^+^	Mean	SEM	Mean	SEM	FL+/Δ2+	Δ2+/FL+	FL+Δ2-/ FL-Δ2+	FL^−^Δ2^+^/ FL^+^Δ2^−^	FL^+^Δ2^+^/ FL^−^Δ2^−^	FL^−^Δ2^−^/ FL^+^Δ2^+^
FOXP3-FL^+^	32.01	4.81	7623.83	2953.90	4.37					
FOXP3-Δ2^+^	7.84	0.67	1486.33	354.48		0.28				
FOXP3-FL^+^Δ2^−^	28.44	4.46	807.17	280.99			11.09			
FOXP3-FL^−^Δ2^+^	4.27	0.78	6816.67	2675.03				0.19		
FOXP3-FL^+^Δ2^+^	3.57	0.45	679.17	131.63					0.06	
FOXP3-FL^−^Δ2^−^	63.70	4.11	11,906.83	2931.52						20.77

**Table 4:** PD-1 and CTLA-4 expression across FOXP3 isoform-defined CD4^+^CD25^+^ T-cell subsets. Mean ± SEM percentages and absolute event counts of CTLA-4/PD-1 expression patterns within FOXP3-FL^+^ and FOXP3-Δ2^+^ CD4^+^CD25^+^ T cells. FL^+^/Δ2^+^ and Δ2^+^/FL^+^ event ratios were calculated for each donor individually and are presented as the mean of donor-specific ratios. FL: full-length Foxp3 isoform; Δ2: exon-2-lacking Foxp3 isoform; SEM: standard error of the mean.

	(FOXP3-FL^+^)		(FOXP3-Δ2^+^)		(FOXP3-FL^+^)		(FOXP3-Δ2^+^)		EVENTS RATIO	
Subpopulation (CD4+CD25+)	% (Mean)	SEM	% (Mean)	SEM	Events (Mean)	SEM	Events (Mean)	SEM	FL^+^/Δ2^+^	Δ2^+^/FL^+^
CTLA-4^+^	0.24	0.06	1.38	0.36	26.33	13.86	22.00	9.35	0.85	2.36
PD-1^+^	2.90	0.37	6.59	0.19	261.50	116.74	99.67	26.21	2.02	0.73
CTLA-4^+^PD-1^−^	0.20	0.04	1.11	0.25	20.33	10.09	16.50	6.03	0.89	2.17
CTLA-4^−^PD-1^+^	2.86	0.35	6.32	0.15	255.50	113.10	94.17	23.36	2.12	0.71
CTLA-4^+^PD-1^+^	0.04	0.02	0.27	0.14	6.00	3.79	5.50	3.33	0.75	0.89
CTLA-4^−^PD-1^−^	96.84	0.39	92.12	0.42	7337.17	2826.95	1367.50	322.25	4.60	0.27

**Table 5:** FOXP3 isoform distribution on CD8^+^ T cells. Mean ± SEM percentages and absolute event counts of FOXP3-FL and FOXP3-Δ2 isoform combinations within CD8^+^ T cells. FL^+^/Δ2^+^ and Δ2^+^/FL^+^ event ratios were calculated for each donor individually and are presented as the mean of donor-specific ratios. FL: full-length Foxp3 isoform; Δ2: exon-2-lacking Foxp3 isoform; SEM: standard error of the mean.

	%		Events		Events Ratio (Mean)					
CD8+	Mean	SEM	Mean	SEM	FL+/Δ2+	Δ2+/FL+	FL+Δ2-/ FL-Δ2+	FL^−^Δ2^+^/ FL^+^Δ2^−^	FL^+^Δ2^+^/ FL^−^Δ2^−^	FL^−^Δ2^−^/ FL^+^Δ2^+^
FOXP3-FL^+^	0.14	0.05	90.83	30.30	0.20					
FOXP3-Δ2^+^	0.80	0.12	507.17	98.71		10.86				
FOXP3-FL^+^Δ2^−^	0.11	0.04	72.67	22.54			0.18			
FOXP3-FL^−^Δ2^+^	0.78	0.13	489.00	98.74				12.62		
FOXP3-FL^+^Δ2^+^	0.03	0.01	18.17	7.96					0.00	
FOXP3-FL^−^Δ2^−^	99.07	0.11	67,385.33	11,047.59						10,453.89

**Table 6:** PD-1 and CTLA-4 expression across FOXP3 isoform-defined CD8+ T-cell subsets. Mean ± SEM percentages and absolute event counts of CTLA-4/PD-1 expression patterns within FOXP3-FL^+^ and FOXP3-Δ2^+^ CD8+ T cells. FL^+^/Δ2^+^ and Δ2^+^/FL^+^ event ratios were calculated for each donor individually and are presented as the mean of donor-specific ratios. FL: full-length Foxp3 isoform; Δ2: exon-2-lacking Foxp3 isoform; SEM: standard error of the mean.

	(FOXP3-FL^+^)		(FOXP3-Δ2^+^)		(FOXP3-FL^+^)		(FOXP3-Δ2^+^)		EVENTS RATIO	
Subpopulation (CD8+)	% (Mean)	SEM	% (Mean)	SEM	Events (Mean)	SEM	Events (Mean)	SEM	FL^+^/Δ2^+^	Δ2^+^/FL^+^
CTLA-4^+^	0.83	0.38	1.32	0.70	1.00	0.52	7.17	4.08	0.08	5.61
PD-1^+^	3.95	2.30	0.91	0.67	2.83	1.35	3.50	2.23	0.76	0.79
CTLA-4^+^PD-1^−^	0.83	0.38	1.32	0.70	1.00	0.52	7.17	4.08	0.08	5.61
CTLA-4^−^PD-1^+^	4.39	2.18	0.91	0.67	2.83	1.35	3.50	2.23	0.76	0.79
CTLA-4^+^PD-1^+^	0.00	0.00	0.00	0.00	0.00	0.00	0.00	0.00	0.00	0.00
CTLA-4^−^PD-1^−^	94.47	2.09	97.55	1.36	86.33	28.29	495.00	96.63	0.20	11.72

**Table 7:** FOXP3 isoform distribution in monocytes. Mean ± SEM percentages and absolute event counts of FOXP3-FL and FOXP3-Δ2 isoform combinations within monocytes. FL^+^/Δ2^+^ and Δ2^+^/FL^+^ event ratios were calculated for each donor individually and are presented as the mean of donor-specific ratios. FL: full-length Foxp3 isoform; Δ2: exon-2-lacking Foxp3 isoform; SEM: standard error of the mean.

	%		Events		Events Ratio (Mean)					
MONOCYTE	Mean	SEM	Mean	SEM	FL+/Δ2+	Δ2+/FL+	FL+Δ2-/ FL-Δ2+	FL^−^Δ2^+^/ FL^+^Δ2^−^	FL^+^Δ2^+^/ FL^−^Δ2^−^	FL^−^Δ2^−^/ FL^+^Δ2^+^
FOXP3-FL^+^	0.22	0.07	121.83	21.97	0.06					
FOXP3-Δ2^+^	3.81	0.77	2637.33	863.11		22.10				
FOXP3-FL^+^Δ2^−^	0.13	0.04	77.67	10.80			0.04			
FOXP3-FL^−^Δ2^+^	3.73	0.76	2593.17	857.80				34.67		
FOXP3-FL^+^Δ2^+^	0.08	0.03	44.17	12.41					0.00	
FOXP3-FL^−^Δ2^−^	96.05	0.78	65,601.00	10,882.71						2564.93

**Table 8:** PD-1 and CTLA-4 expression across FOXP3 isoform-defined monocyte subsets. Mean ± SEM percentages and absolute event counts of CTLA-4/PD-1 expression patterns within FOXP3-FL^+^ and FOXP3-Δ2^+^ monocytes. FL^+^/Δ2^+^ and Δ2^+^/FL^+^ event ratios were calculated for each donor individually and are presented as the mean of donor-specific ratios. FL: full-length Foxp3 isoform; Δ2: exon-2-lacking Foxp3 isoform; SEM: standard error of the mean.

	(FOXP3-FL^+^)		(FOXP3-Δ2^+^)		(FOXP3-FL^+^)		(FOXP3-Δ2^+^)		EVENTS RATIO	
Subpopulation (MONOCYTE)	% (Mean)	SEM	% (Mean)	SEM	Events (Mean)	SEM	Events (Mean)	SEM	FL^+^/Δ2^+^	Δ2^+^/FL^+^
CTLA-4^+^	0.64	0.32	0.51	0.10	0.67	0.33	14.67	5.55	0.06	17.83
PD-1^+^	1.78	0.52	0.94	0.34	2.00	0.58	18.67	5.30	0.15	9.63
CTLA-4^+^PD-1^−^	0.64	0.32	0.48	0.11	0.67	0.33	16.20	6.06	0.08	16.83
CTLA-4^−^PD-1^+^	1.78	0.52	0.91	0.33	2.00	0.58	18.00	5.17	0.15	9.20
CTLA-4^+^PD-1^+^	0.00	0.00	0.03	0.02	0.00	0.00	0.67	0.33	0.00	0.00
CTLA-4^−^PD-1^−^	95.81	0.49	98.44	0.32	117.17	21.62	2601.33	854.46	0.06	22.77

**Table 9:** FOXP3 isoform distribution in neutrophils. Mean ± SEM percentages and absolute event counts of FOXP3-FL and FOXP3-Δ2 isoform combinations within neutrophils. FL^+^/Δ2^+^ and Δ2^+^/FL^+^ event ratios were calculated for each donor individually and are presented as the mean of donor-specific ratios. FL: full-length Foxp3 isoform; Δ2: exon-2-lacking Foxp3 isoform; SEM: standard error of the mean.

	%		Events		Events Ratio (Mean)					
NEUTROPHIL	Mean	SEM	Mean	SEM	FL+/Δ2+	Δ2+/FL+	FL+Δ2-/ FL-Δ2+	FL^−^Δ2^+^/ FL^+^Δ2^−^	FL^+^Δ2^+^/ FL^−^Δ2^−^	FL^−^Δ2^−^/ FL^+^Δ2^+^
FOXP3-FL^+^	0.16	0.06	853.17	155.02	0.07					
FOXP3-Δ2^+^	7.66	3.82	47,422.00	30,292.90		50.88				
FOXP3-FL^+^Δ2^−^	0.10	0.03	603.33	96.50			0.07			
FOXP3-FL^−^Δ2^+^	7.60	3.80	47,172.17	30,208.99				96.25		
FOXP3-FL^+^Δ2^+^	0.06	0.03	249.83	116.79					0.00	
FOXP3-FL^−^Δ2^−^	92.24	3.83	66,4804.00	126,147.29						11,587.30

**Table 10:** PD-1 and CTLA-4 expression across FOXP3 isoform-defined neutrophil subsets. Mean ± SEM percentages and absolute event counts of CTLA-4/PD-1 expression patterns within FOXP3-FL^+^ and FOXP3-Δ2^+^ neutrophils. FL^+^/Δ2^+^ and Δ2^+^/FL^+^ event ratios were calculated for each donor individually and are presented as the mean of donor-specific ratios. FL: full-length Foxp3 isoform; Δ2: exon-2-lacking Foxp3 isoform; SEM: standard error of the mean.

	(FOXP3-FL^+^)		(FOXP3-Δ2^+^)		(FOXP3-FL^+^)		(FOXP3-Δ2^+^)		EVENTS RATIO	
Subpopulation (NEUTROPHIL)	% (Mean)	SEM	% (Mean)	SEM	Events (Mean)	SEM	Events (Mean)	SEM	FL^+^/Δ2^+^	Δ2^+^/FL^+^
CTLA-4^+^	0.76	0.15	0.14	0.02	6.67	2.39	54.00	26.93	0.38	10.68
PD-1^+^	0.54	0.12	0.07	0.03	5.17	1.83	36.33	20.80	0.79	7.36
CTLA-4^+^PD-1^−^	0.72	0.16	0.14	0.02	6.33	2.43	54.00	26.93	0.34	11.03
CTLA-4^−^PD-1^+^	0.50	0.11	0.07	0.03	4.83	1.82	36.33	20.80	0.69	7.40
CTLA-4^+^PD-1^+^	0.04	0.03	0.00	0.00	0.33	0.21	0.00	0.00	0.00	0.00
CTLA-4^−^PD-1^−^	98.67	0.26	99.76	0.04	840.83	151.23	47,318.17	30,240.40	0.07	51.38

## Data Availability

The original contributions presented in this study are included in the article/Supplementary Material.

## Supplementary Materials

The following supporting information can be downloaded at https://www.mdpi.com/article/10.3390/biom16070948/s1, Representative FMO gates for CD4+ subpopulations and for CD8+ T cells, monocytes, and neutrophils are shown in Supplementary Figures S1 and S2, respectively. Supplementary Figure S1. FMO controls for CD4^+^ T-cell subpopulations. Representative fluorescence-minus-one (FMO) controls used to define positivity thresholds for each marker. FOXP3-FL and FOXP3-Δ2 FMO controls are shown within the CD4^+^CD25^+^ and CD4^+^CD25^−^ gates, respectively. CTLA-4 and PD-1 FMO controls were acquired and gated on the total CD4^+^ population and applied uniformly to both CD4^+^CD25^+^ and CD4^+^CD25^−^ subsets. Dashes (–) indicate that no independent FMO control was required for that marker–population combination. Gates shown are representative of one donor and were applied consistently across all six donors. Supplementary Figure S2. FMO controls for CD8+T cels, monocyte and neutrophil. CTLA-4 and PD-1 FMO controls were performed within each specific cell population (CD8^+^ T cells, monocytes, and neutrophils). Given the inherently low expression of these markers in these populations under basal conditions, the resulting FMO gates provide highly stringent positivity thresholds, minimizing the risk of false-positive assignment.

## Abbreviations

CTLA-4	Cytotoxic T-lymphocyte-associated protein 4
EDTA	Ethylenediaminetetraacetic acid
FBS	Fetal bovine serum
FMO	Fluorescence-minus-one
FOXP3	Forkhead box P3
FOXP3-FL	Full-length FOXP3 isoform
FOXP3-Δ2	Exon-2-deficient FOXP3 isoform
FSC-A	Forward scatter area
FSC-H	Forward scatter height
GRCh38	Genome Reference Consortium Human Build 38
IL-2	Interleukin-2
IPEX	Immunodysregulation, polyendocrinopathy, enteropathy, X-linked syndrome
mRNA	Messenger RNA
NK	Natural killer
PBMC	Peripheral blood mononuclear cell
PBS	Phosphate-buffered saline
PD-1	Programmed cell death protein 1
PD-L1	Programmed death-ligand 1
SEM	Standard error of the mean
SSC-A	Side scatter area
TCR	T-cell receptor
Treg	Regulatory T cell
