# Supplementary material for: First Comprehensive Analysis of Full-Length and Δ2 Foxp3 Isoforms Distribution in PBMCs from Healthy Volunteers
Source: Biomolecules. 2026 Jun 25;16(7):948. doi: 10.3390/biom16070948 (PMC13406201; doi:10.3390/biom16070948)
Supplement: Supplementary file 1 [file biomolecules-16-00948-s001.zip › biomolecules-4350008-supplementary.pdf]

<table>
<tr><th></th><th>CD25</th><th>FOXP3-FL</th><th>FOXP3-Δ2</th><th>CTLA-4</th><th>PD-1</th></tr>
<tr><td>CD4</td><td>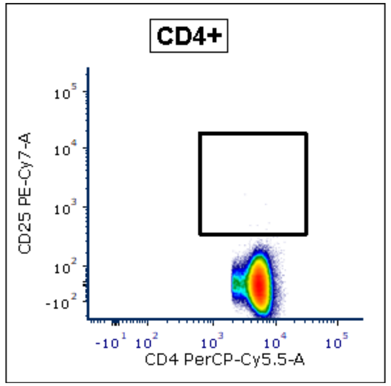</td><td>–</td><td>–</td><td>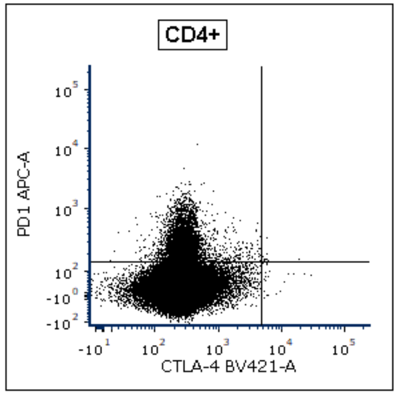</td><td>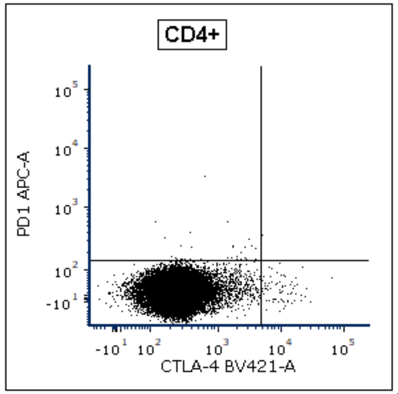</td></tr>
<tr><td>CD4⁺CD25⁻</td><td>–</td><td>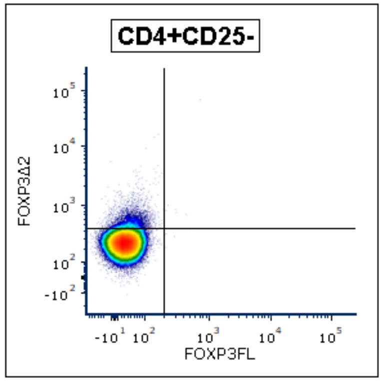</td><td>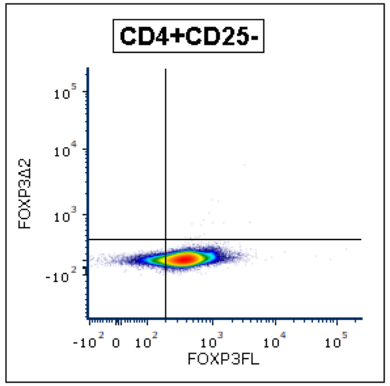</td><td>–</td><td>–</td></tr>
<tr><td>CD4⁺CD25⁺</td><td>–</td><td>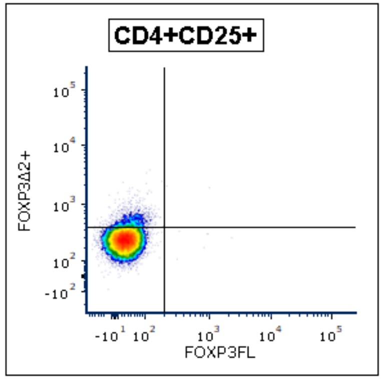</td><td>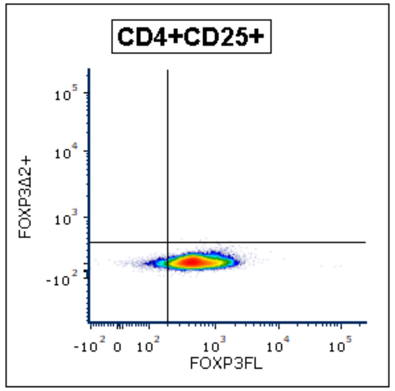</td><td>–</td><td>–</td></tr>
</table>

Supplementary Figure S1. FMO controls for $CD4^+$ T-cell subpopulations. Representative fluorescence-minus-one (FMO) controls used to define positivity thresholds for each marker. FOXP3-FL and FOXP3-Δ2 FMO controls are shown within the $CD4^+CD25^+$ and $CD4^+CD25^-$ gates, respectively. CTLA-4 and PD-1 FMO controls were acquired and gated on the total $CD4^+$ population and applied uniformly to both $CD4^+CD25^+$ and $CD4^+CD25^-$ subsets. Dashes (–) indicate that no independent FMO control was required for that marker–population combination. Gates shown are representative of one donor and were applied consistently across all six donors.

	CD25	FOXP3-FL	FOXP3-Δ2	CTLA-4	PD-1
**CD8+**	–	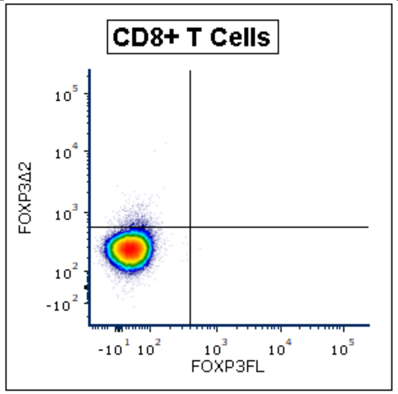	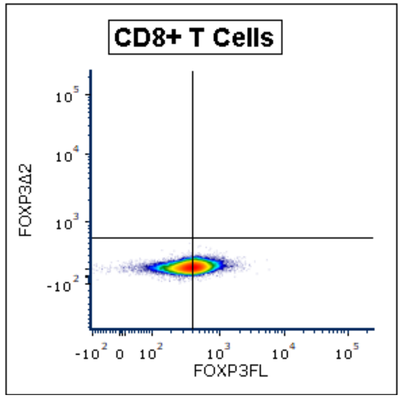	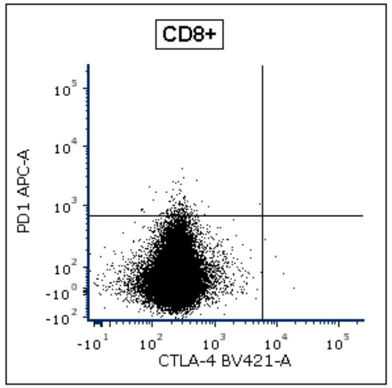	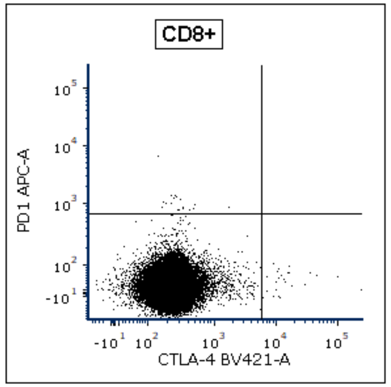
**MONOCYTE**	–	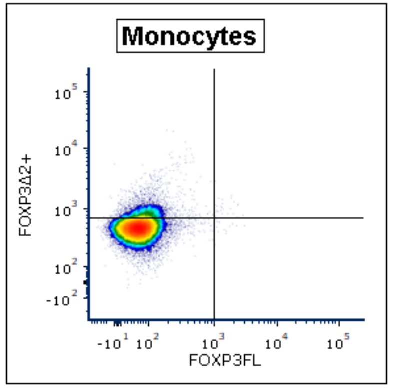	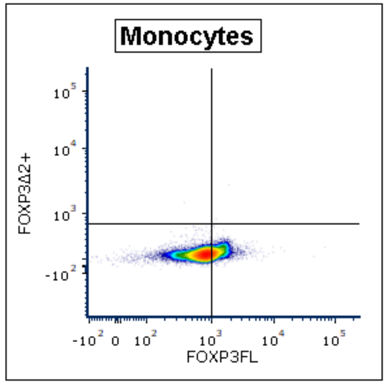	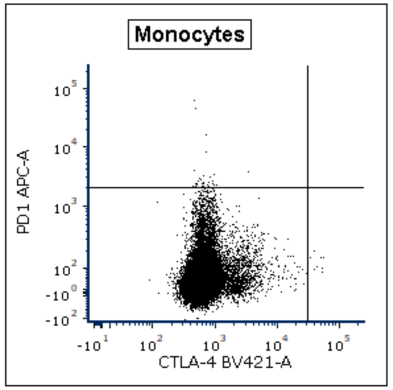	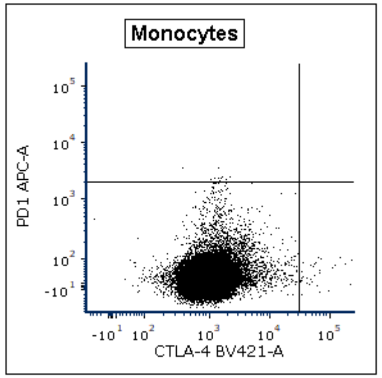
**NEUTROPHIL**	–	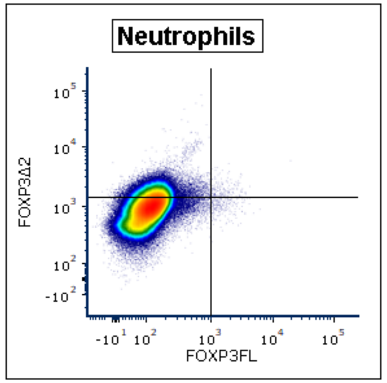	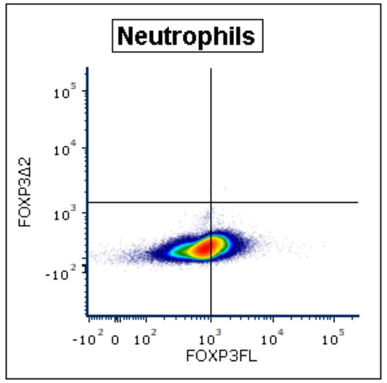	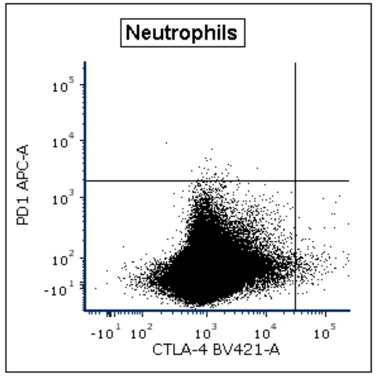	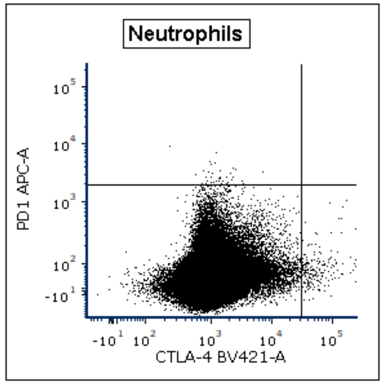

Supplementary Figure S2. FMO controls for CD8$^+$ T celss, monocyte and neutrophil CTLA-4 and PD-1 FMO controls were performed within each specific cell population (CD8$^+$ T cells, monocytes, and neutrophils). Given the inherently low expression of these markers in these populations under basal conditions, the resulting FMO gates provide highly stringent positivity thresholds, minimizing the risk of false-positive assignment.
